# Yoshimura-origami Based Earthworm-like Robot With 3-dimensional Locomotion Capability

**DOI:** 10.3389/frobt.2021.738214

**Published:** 2021-08-20

**Authors:** Qiwei Zhang, Hongbin Fang, Jian Xu

**Affiliations:** ^1^ School of Aerospace Engineering and Applied Mechanics, Tongji University, Shanghai, China; ^2^ Institute of AI and Robotics, Fudan University, Shanghai, China; ^3^ Engineering Research Center of AI & Robotics, Ministry of Education, Fudan University, Shanghai, China; ^4^ Shanghai Engineering Research Center of AI & Robotics, Fudan University, Shanghai, China

**Keywords:** bio-inspired robot, origami kinematics, origami robot, peristaltic locomotion, locomotion gait

## Abstract

Earthworm-like robots have received great attention due to their prominent locomotion abilities in various environments. In this research, by exploiting the extraordinary three-dimensional (3D) deformability of the Yoshimura-origami structure, the state of the art of earthworm-like robots is significantly advanced by enhancing the locomotion capability from 2D to 3D. Specifically, by introducing into the virtual creases, kinematics of the non-rigid-foldable Yoshimura-ori structure is systematically analyzed. In addition to exhibiting large axial deformation, the Yoshimura-ori structure could also bend toward different directions, which, therefore, significantly expands the reachable workspace and makes it possible for the robot to perform turning and rising motions. Based on prototypes made of PETE film, mechanical properties of the Yoshimura-ori structure are also evaluated experimentally, which provides useful guidelines for robot design. With the Yoshimura-ori structure as the skeleton of the robot, a hybrid actuation mechanism consisting of SMA springs, pneumatic balloons, and electromagnets is then proposed and embedded into the robot: the SMA springs are used to bend the origami segments for turning and rising motion, the pneumatic balloons are employed for extending and contracting the origami segments, and the electromagnets serve as anchoring devices. Learning from the earthworm’s locomotion mechanism--retrograde peristalsis wave, locomotion gaits are designed for controlling the robot. Experimental tests indicate that the robot could achieve effective rectilinear, turning, and rising locomotion, thus demonstrating the unique 3D locomotion capability.

## Introduction

In recent years, earthworm-like locomotion robots have received great attention due to their excellent mobility in narrow space and unstructured environments, enabling many potential applications such as pipe cleaning (Tanise et al., 2017; Fang et al., 2014; Adams et al., 2018), gastrointestinal examination (Wang et al., 2008; Rodríguez et al., 2006), and battlefield surveillance (Fields et al., 2009). The superior locomotive ability of the earthworm-like robots mainly originates from the specially designed robot structure and the elaborated control strategies, where the former is inspired by the earthworm’s unique morphology characteristics, and the latter is a representation of the earthworm’s fundamental locomotion mechanism. Specifically, in terms of the earthworm’s morphology merits, the following three aspects are mainly emphasized: *1*) the earthworm’s body consists of a large number of independent working segments separated by septa (Edwards and Bohlen, 1996); *2*) each segment possesses circular and longitudinal muscles that work antagonistically to each other (Alexander, 2013), giving rise to interrelated radial and axial deformations of the segment; *3*) the bulk of segments have bristle-like setae that can help to anchor parts of the body to the working media during movement (Edwards and Bohlen, 1996). As a consequence, most earthworm-like locomotion robots are designed to be metameric (Fang et al., 2014; Omori et al., 2009; Omori et al., 2008; Isaka et al., 2019; Song et al., 2016), with each robot segment being equipped with antagonistic actuators (Fang et al., 2014; Omori et al., 2009; Omori et al., 2008; Isaka et al., 2019; Song et al., 2016) and anchoring mechanisms (Ge et al., 2017; Daltorio et al., 2013; Tanaka et al., 2012; Horchler et al., 2015). In terms of the locomotion mechanism, by coordinating the deformations of the earthworm’s body segments, a peristalsis wave can be generated, which propagates along the earthworm’s body in the opposite direction to its movement (Quillin, 1999). It has been demonstrated that by alternating the characteristics of the wave, the earthworm could adjust its locomotion mode to adapt to different surroundings (Omori et al., 2009; Ge et al., 2017). Accordingly, learning from the earthworm’s retrograde peristalsis wave, different types of robotic control have been proposed for achieving rectilinear/planar locomotion; the control strategies can be either discrete (e.g., gait tables (Fang et al., 2015a)) or continuous (e.g., phase coordination (Fang et al., 2015b) and wave controller (Boxerbaum et al., 2012)), and their effectiveness have been experimentally verified.

Based on the compliance of the robot’s constituent materials (Trivedi et al., 2008; Majidi, 2014), the existing robot prototypes can be classified into two categories: rigid earthworm-like robots and soft earthworm-like robots. For the former, the robot body is always composed of rigid parts, such as acrylic plates, rigid resin skeletons, and spring-steel belts, etc. (Fang et al., 2014; Zarrouk and Shoham, 2012). In general, a rigid robot segment can be manufactured and assembled relatively easily; it could adapt to various types of actuators (e.g., shape memory alloys (SMA) (Kim et al., 2006) and servomotors (Fang et al., 2014; Kim et al., 2006)), while its deformability is often limited. For the latter, the robot body is always made up of continuously deformable elements (e.g. coupled cables (Daltorio et al., 2013; Kandhari et al., 2018)) or soft/extensible materials (e.g. rubber and silicone (Harigaya et al., 2013; Calderón et al., 2019)). To achieve effective worm-like locomotion, pneumatic actuators (Deng et al., 2020; Tang et al., 2020) and active materials driven by external physical fields (Hu et al., 2018) have been extensively employed, in part because they are very compatible with the soft robot body. The advantage of soft robots is that they can exhibit high continuous deformations and locomotion agility. However, precise control of the soft body is still a big challenge, since the constitutive relations of the soft material and soft actuators have not been well understood. Apart from the inherent pros and cons of the rigid and soft earthworm-like robots, they all meet the challenges in terms of weight and size, which calls for further innovation in robot design.

Despite the abovementioned progresses in understanding and mimicking the earthworm’s morphological characteristics, another important concern of earthworm-like robots is the diversity of locomotion modes. As the first step, rectilinear locomotion has attracted the majority of attention. For example, based on servomotor (Fang et al., 2015a), SMA (Kim et al., 2006), or cable actuators (Boxerbaum et al., 2012) and by prescribing the gait based on earthworms’ peristalsis waves, rectilinear earthworm-like robotic locomotion has been realized in both pipe and open environment. However, rectilinear robotic locomotion, generally, is not sufficient in applications. Note that in nature, earthworms can also perform planar and spatial locomotion. By shortening the longitudinal muscles at one side and stretching the longitudinal muscles at the other side, the earthworm could bend its body and make a turn or make a rise. Inspired by such biology observation and by integrating more independent actuators into the robot design, the locomotion mode has been significantly enriched. For example, with three independent servomotor arms in each segment, Omori et al. developed a four-segment earthworm-like robot that is able to turn on a plane surface and move upward/downward in a vertical pipe (Omori et al., 2009). Similarly, *via* embedding more actuators into the robot, planar locomotion has also been verified to be feasible in the CMMWorm-S robot (Kandhari et al., 2018), the metameric robot (Zhan et al., 2019), and the Meshworm (Seok et al., 2013). In these successful prototypes, antagonistic axial and radial deformations of the robot segment/part play an important role in achieving locomotion. Segments/parts without such antagonistic deformability can also be utilized for robot design, providing that additional bristle structures (e.g. microspine and clamping devices (Fang D. et al., 2018; Yang et al., 2019)) are embedded to acquire anchoring effect.

Further advancing the locomotion capability of the earthworm-like robot from 2-dimensional (2D) to 3-dimensional (3D) is a nice expectation. However, the development remains stagnant; effective designs and prototypes of earthworm-like spatial locomotion robots have not been reported. One of the reasons lies in that there is a lack of structure that has the advantages of strong 3D deformability, lightweight, and good adaptation to actuators. Origami, originally a recreational art, is a promising platform to tackle the above bottleneck problem due to its limitless design space, extraordinary deformability, and unique reconfigurability. The essence of origami is to construct complex 3D shapes *via* folding 2D flat sheets following elaborate crease patterns. Even with rigid-folding assumption, the obtained 3D origami structure could exhibit significant deformations by rotating the rigid facets with respect to the creases, which is entirely different from the conventional mechanisms that rely on rigid linkages and movable joints to deform. Moreover, based on folding techniques, thin and weak materials, such as paper and plastic films, become candidates for robot fabrication, making the obtained origami robots extremely light in weight. Particularly, with the introduction of folds, the origami robots would still have sufficient strength and stiffness. In addition, originated from the nonlinear folding kinematics, certain origami structures are well-known for their unorthodox mechanical properties (Li et al., 2019), including negative Poisson’s ratio (Yasuda and Yang, 2015), stiffness re-programmability (Silverberg et al., 2014), structural multistability (Fang et al., 2017a; Li and Wang, 2015, Zhang et al., 2020, Wu et al., 2020), and self-locking (Fang H. et al., 2018), etc.; they provide brand new possibilities for developing robots with novel or enhanced functionalities. With origami techniques, rather than 3D modeling and shaping, design and fabrication of 3D structures can be finished in the 2D realm. The existing 2D design tools and a wide range of commercial 2D fabrication techniques (lithography, laser machining, and basic chemical etching, etc.) could significantly reduce the cost and shorten the production cycle, and meanwhile, remain high machining precision (Onal et al., 2015; Deng and Chen, 2015, Fang et al., 2017b). Such merits also bring exciting opportunities to robot design and fabrication. Considering the similarities in deformation between the earthworm’s body segment and certain origami structures, serval origami-based earthworm-like robots have been proposed to overcome the drawbacks of conventional designs. For example, Fang et al. designed an earthworm-like robot based on the origami ball structure (Fang et al., 2017a). With a positive Poisson’s ratio, the origami ball could exhibit antagonistically-coupled axial and radial deformations, which well mimic the earthworms’ morphology characteristics. Bhovad et al. (Bhovad et al., 2019) and Pagano et al. (Pagano et al., 2017) employed the Kresling origami structure and setae-like feet to achieve peristaltic locomotion, in which the inherent structural bistability has been exploited for fast actuation and easy control.

Note that for these two origami robot prototypes, 1D rectilinear locomotion is the main purpose to achieve. By incorporating two Kresling origami structures in the robot, 2D planar locomotion is also possible (Pagano et al., 2017). Endowing the origami-based earthworm-like robot with expecting 3D spatial locomotion capability is an important direction to explore, while related efforts have not been made. In this research, to advance the state of the art, a new type of Yoshimura-ori-based earthworm-like robot with unique 3D spatial locomotion capability is developed. This is because the Yoshimura-ori structure possesses excellent axial and bending deformability, which can be exploited to break through the current limitations in achieving turning and rising motions in earthworm-like robots. First, to evaluate the deformability of a single robot segment and to predict the reachable workspace, geometry and kinematics of the Yoshimura-ori structure are systematically examined. Mechanical properties of the Yoshimura-ori structure are also evaluated experimentally, which offers useful guidelines for designing actuation. Using the Yoshimura-ori structure as the skeleton, the SMA spring and pneumatic balloon as the hybrid actuation, and the electromagnet as the anchoring device, an earthworm-like robot is designed and prototyped. Based upon the adopted hybrid actuation and anchoring mechanisms and by mimicking the earthworm’s retrograde peristalsis wave, a gait control strategy is also proposed for locomotion control. Experimental tests indicate that the robot could perform effective rectilinear, turning, and rising locomotion, thus successfully expanding the locomotion ability from 2D to 3D.

## Design and Prototype

**FIGURE 1:**
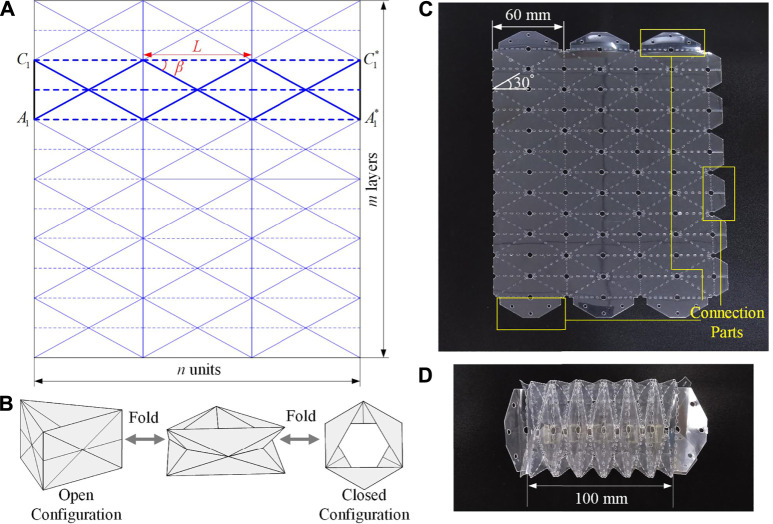
Design and prototype of the Yoshimura-ori structure. **(A)** 2D crease pattern of the 3×6 Yoshimura-ori. By overlapping points $A_{1}$, $C_{1}$ with $A_{1}^{*}$, $C_{1}^{*}$, a Yoshimura-ori structure can be obtained. **(B)** Folding motion between the open configuration and the closed configuration of a single-layer Yoshimura-ori structure. **(C)** The laser-machined 2D sheet of a 3×6 Yoshimura-ori made of PETE film. **(D)** The obtained six-layer Yoshimura-ori structure.

Figure 1A display the flat crease pattern of the Yoshimura-ori, which can be uniquely defined by four parameters, *n*, *m*, *L*, and *β*, denoting the number of the basal rectangles, the number of layers, the length of each basal rectangle, and the angle between the diagonal and the length side, respectively. The solid and dashed lines denote the “mountain” and “valley” crease of the pattern. For a single layer, by overlapping points $A_{1}$, $C_{1}$ with$A_{1}^{*}$, $C_{1}^{*}$, a Yoshimura-ori structure in the open configuration can be obtained. By applying a force on the top of the Yoshimura-ori structure, it will be gradually contracted to its closed configuration, shown in Figure 1B. The transition from the open configuration to the closed configuration is not rigid-foldable (to be discussed in Section 3); rather, elastic deformations of certain facets are necessary to complete the transformation. To ensure that there is no elastic deformation at the closed configuration, the following geometric constraint has to be satisfied

$$\beta =90^{\circ }-\frac{\left(n-1\right)\cdot 180^{\circ }}{2n}.$$ (1)

Diverse polyester materials can be used for making origami structures, such as polyether ether ketone (PEEK), polytetrafluoroethylene (PTFK), polyethylene terephthalate (PETE), and polyimide. Among them, PETE film is selected because it can be easily cut, folded, and has a relatively higher softening temperature around $150^{\circ }\text{C}$ (higher than the heating temperature of SMA). Laser-based machining technology is adopted to cut and pattern the flat PETE sheet owing to its efficiency, precision, convenience, and low cost. Figure 1C shows the laser-machined 2D crease pattern of a 3 × 6 Yoshimura-ori made of PETE film. The length L of the constituent rectangle is 60 mm, and the angle β is $30^{\circ }$. The creases are perforated to some extent to weaken the bending stiffness of both mountain and valley creases so that they are flexible for folding. Small holes are cut at the vertices where multiple folds intersect to reduce or eliminate the possible stress concentration. In addition, for connecting purposes, additional connections parts are added on the left, top, and bottom of the crease pattern (shown in the yellow box in Figure 1C). The laser cutting machine can produce the 2D Yoshimura-ori pattern in about 5 min, and it costs about 30 min to fold and paste the sheet into a 3D Yoshimura-ori structure (Figure 1D). In what follows, all theoretical and experimental studies are based on this Yoshimura-ori structure.

## Kinematic Analyses

**FIGURE 2:**
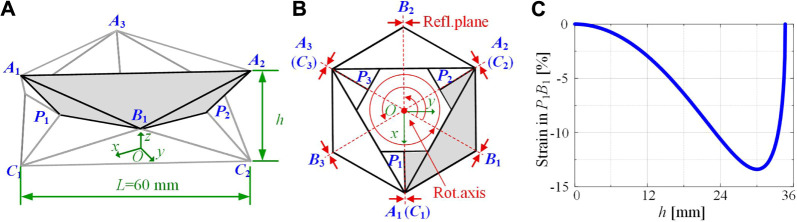
A single-layer Yoshimura-ori structure and the theoretical strain change of the crease $\bar{P_{1}B_{1}}$. **(A)** A single-layer Yoshimura-ori structure. The shaded area represents a rectangle “unit” to be studied. **(B)** Top-down view of the structure. The origin of the O-xyz coordinate system locates at the centroid of the equilateral triangle $\bar{C_{1}C_{2}C_{3}}$, the x-axis is chosen to intersect vertex $C_{1}$, the z-axis is the rotational axis that is perpendicular to the plane $\bar{C_{1}C_{2}C_{3}}$, and the y-axis follows the right-hand rule convention. The reflectional and rotational symmetry of the structure is also indicated. **(C)** Theoretical strain in *P*_1_ *B*_1_ as a function of the height h.

In this section, the folding kinematics of the Yoshimura-ori structure is studied in detail, with major focuses on axial and bending deformability. Assuming that the deformation of each layer is identical, a single-layer Yoshimura-ori structure is taken out for study (Figure 2A). After understanding the folding behavior of a single-layer structure, the reachable workspace of a multi-layer structure is examined, which is an important index for robot design. Note that Yoshimura-ori structure has also been exploited for pneumatic actuation (Paez et al., 2016), energy absorption (Yang et al., 2016), and Barrel Vault (Cai et al., 2015), however, a theoretical framework of folding kinematics has not been developed.

### Axial Kinematics

We start with analyzing the degree of freedom (DOF) of a single-layer Yoshimura-ori structure. With rigid-folding assumption, the whole structure can be equivalent to a space truss frame. Hence, the DOF of a single-layer Yoshimura-ori structure can be determined by (Saito et al., 2015):

$$N_{\text{DOF}}=3\cdot N_{\text{vertex}}-N_{\text{crease}}-6,$$ (2)

where $N_{\text{vertex}}$ is the total number of vertex, $N_{\text{crease}}$ is the total number of creases. For a single layer Yoshimura-ori structure, $N_{\mathrm{vertex}}=12$ and *N*_crease_ = 30; hence, the DOF of the Yoshimura-ori structure is zero, which indicates that it cannot be transformed from the open configuration to the closed configuration by rigid folding. To achieve such transformation, facet bending is a necessity.

To understand the folding kinematics of the Yoshimura-ori structure, a cartesian coordinate system *O-xyz* is set, with the origin locating at the centroid of the equilateral triangle $\bar{C_{1}C_{2}C_{3}}$ (its position and orientation remain fixed during folding). Considering the $C_{3}$rotational symmetry (Figure 2B) and the top-down reflectional symmetry (i.e., symmetric with respect to the plane $\bar{B_{1}B_{2}B_{3}}$) of a single layer Yoshimura-ori structure, examining a rectangle “unit” $\bar{A_{1}A_{2}P_{2}P_{1}}$ is sufficient to determine the folding motion. The positions of each vertex are not independent, instead, they are constrained by the geometric lengths of the creases “$\bar{A_{1}A_{2}}$,” “$\bar{A_{1}P_{1}}$,” “$\bar{A_{2}P_{2}}$,” “$\bar{A_{1}B_{1}}$,” and “$\bar{A_{2}B_{1}}$,” which give rise to the following constraint equations:

$$l_{\bar{A_{1}A_{2}}}=L,l_{\bar{A_{1}P_{1}}}=l_{\bar{A_{2}P_{2}}}=\frac{\sqrt{3}L}{6},l_{\bar{A_{1}B_{1}}}=l_{\bar{A_{2}B_{1}}}=\frac{\sqrt{3}L}{2}.$$ (3)

As mentioned early, folding of the Yoshimura-ori structure calls for elastic deformations of certain facets or creases. To understand the folding behavior, the lengths of the crease $\bar{P_{1}B_{1}}$ and $\bar{P_{2}B_{1}}$ (i.e., $\left\|l_{\bar{P_{1}B_{1}}}\right\|$ and $\left\|l_{P_{2}B_{1}}\right\|$) are assumed to be mathematically variable during the whole folding process. Due to the reflection symmetry of the structure about the plane $\bar{A_{1}C_{1}B_{3}}$, $\left\|l_{\bar{P_{1}B_{1}}}\right\|=\left\|l_{\bar{P_{2}B_{1}}}\right\|$. This provides the structure with an additional DOF to transform between the closed and the open configurations. With such a conceptual simplification, the deformation of the structure can be quantified *via* a single parameter. Here, we choose the height of a single layer Yoshimura-ori structure (Figure 2A), denoted by *h*, as the independent variable, because it is related to the actuation in the robot prototype (to be discussed in Section 5). Hence, the position of each vertex can be derived:

$$\begin{array}{l} A_{1}=\left[\sqrt{3}L/3,0,h\right],\\ A_{2}=\left[-\sqrt{3}L/6,L/2,h\right],\\ B_{1}=\left[\frac{1}{2}\left(\frac{\sqrt{3}L}{6}+\sqrt{\frac{L^{2}-3h^{2}}{12}}\right),\frac{\sqrt{3}}{2}\left(\frac{\sqrt{3}L}{6}+\sqrt{\frac{L^{2}-3h^{2}}{12}}\right),\frac{h}{2}\right],\\ P_{1}=\left[\left(\frac{\sqrt{3}L}{3}-\sqrt{\frac{L^{2}-3h^{2}}{12}}\right),0,\frac{h}{2}\right],\\ P_{2}=\left[-\frac{1}{2}\left(\frac{\sqrt{3}L}{3}-\sqrt{\frac{L^{2}-3h^{2}}{12}}\right),\frac{\sqrt{3}}{2}\left(\frac{\sqrt{3}L}{3}-\sqrt{\frac{L^{2}-3h^{2}}{12}}\right),\frac{h}{2}\right]. \end{array}$$ (4)

Based on this, the distance between $P_{1}$ and $B_{1}$ can be calculated, and accordingly, the theoretical strain of the crease $\bar{P_{1}B_{1}}$ can be obtained. Figure 2C displays the evolution of the strain with respect to the height h. When h=0 mm and h=34.64 mm, the strain is zero, indicating that the structure has no elastic deformation at either the open (h=34.64 mm) or the closed (h=0 mm) configuration. With the increase of h from 0 to the maximum, the strain is non-zero and remains negative, suggesting that the crease has to be shortened to achieve the transformation from the closed state to the open state.

**FIGURE 3:**
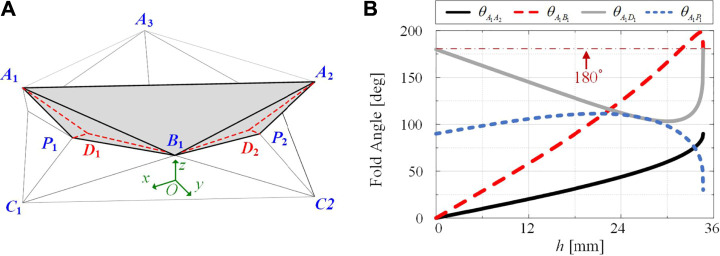
Kinematics of a single-layer Yoshimura-ori structure with virtual creases. **(A)** Introduction of virtual creases to account for facet bending. With virtual creases, panels $\bar{A_{1}P_{1}D_{1}}$, $\bar{A_{1}D_{1}B_{1}}$, $\bar{A_{2}D_{2}B_{1}}$, and $\bar{A_{2}P_{2}D_{2}}$ are treated as rigid during folding. **(B)** Dihedral angles as functions of the height h.

While the strain could effectively illustrate the non-rigid-folding of the structure, shortening of the free length $\bar{P_{1}B_{1}}$ does not agree with the actual deformation of a single-layer Yoshimura-ori prototype. To better describe the folding behavior, facet bending is taken into account. This is because the primary deformation mode of a facet is bending, rather than stretching. Specifically, to account for the bending behavior, a “virtual crease” is introduced into the bending facet, which represents the “hidden” DOF related to the out-of-plane bending deformation. According to experimental observations, two virtual creases, $\bar{A_{1}D_{1}}$ and $\bar{A_{2}D_{2}}$, are placed, as shown in Figure 3A. Hence, facet $\bar{A_{1}B_{1}P_{1}}$ is divided into two rigid triangular panels $\bar{A_{1}P_{1}D_{1}}$ and $\bar{A_{1}D_{1}B_{1}}$; $\bar{A_{2}B_{1}P_{2}}$ is also divided into two rigid triangular planes $\bar{A_{2}D_{2}B_{1}}$ and $\bar{A_{2}P_{2}D_{2}}$. Noting that panels $\bar{A_{2}D_{2}B_{1}}$ and $\bar{A_{2}P_{2}D_{2}}$ are mirror reflections of panels $\bar{A_{1}P_{1}D_{1}}$ and $\bar{A_{1}D_{1}B_{1}}$, hence, in what follows, only the virtual crease $\bar{A_{1}D_{1}}$ and the associated panels $\bar{A_{1}P_{1}D_{1}}$ and $\bar{A_{1}D_{1}B_{1}}$are considered.

When the Yoshimura-ori structure is axially contracted or extended, the position of vertex $A_{1}$ is still determined by Equation 4, while the newly introduced vertex $D_{1}$ is free to move, providing that the following geometric constraints are satisfied:

$$l_{\bar{P_{1}D_{1}}}=\text{constant},\;l_{\bar{D_{1}B_{1}}}=\text{constant},\;l_{\bar{P_{1}D_{1}}}+l_{\bar{D_{1}B_{1}}}=\frac{L}{2},$$ (5a)

$$l_{\bar{A_{1}D_{1}}}=\sqrt{l_{\bar{P_{1}D_{1}}}^{2}+l_{\bar{A_{1}P_{1}}}^{2}}=\text{constant}.$$ (5b)

Equation 5a indicates that the crease lengths corresponding to $\bar{P_{1}D_{1}}$ and $\bar{D_{1}B_{1}}$ remain constant during folding, and their sum equals the initial distance between vertices $P_{1}$ and $B_{1}$ (i.e., L/2). Here, the lengths of creases $\bar{P_{1}D_{1}}$ and $\bar{D_{1}B_{1}}$ are set based on observations. Equation 5b indicates that the length of the virtual crease $\bar{A_{1}D_{1}}$ remains constant during folding, which is determined from the open or closed configuration.

Combining Equations 4, 5, the coordinates of all vertices can be determined at each step of folding *via* numerical methods. Further, the dihedral angles between adjacent facets can be calculated based on the law of cosines, in which the normal vectors of the associated facets are used. For example, the dihedral angle at the crease $\bar{A_{1}B_{1}}$, i.e., $\theta_{\bar{A_{1}B_{1}}}$, can be calculated *via*

$$\vec{V_{1}}=\vec{D_{1}B_{1}}\times \vec{B_{1}A_{1}},$$ (6a)

$$\vec{V_{2}}=\vec{B_{1}A_{1}}\times \vec{A_{1}A_{2}},$$ (6b)

$$\theta_{\bar{A_{1}B_{1}}}=\cos^{-1}\left(\frac{\vec{V_{1}}\cdot \vec{V_{2}}}{\left\|\vec{V_{1}}\right\|\cdot \left\|\vec{V_{2}}\right\|}\right),$$ (6c)

Where $\vec{V_{1}}$ and $\vec{V_{2}}$ are normal vectors of panels $\bar{A_{1}D_{1}B_{1}}$ and $\bar{A_{1}D_{1}B_{1}}$, respectively, ‖•‖ denotes the Euclidean length. Note that there are four dihedral angles that are related to the studied panels $\bar{A_{1}P_{1}D_{1}}$ and $\bar{A_{1}D_{1}B_{1}}$, they are: $\theta_{\bar{A_{1}B_{1}}}$, $\theta_{\bar{A_{1}D_{1}}}$, $\theta_{\bar{A_{1}P_{1}}}$, and $\theta_{\bar{A_{1}A_{2}}}$ (defined as the dihedral angle between the panel $\bar{A_{1}A_{2}B_{1}}$ and the plane $\bar{A_{1}A_{2}A_{3}}$). Figure 3B displays the four dihedral angles as functions of the height h. It reveals that when h=0 mmand h=34.64 mm, the virtual fold angle $\theta_{\bar{A_{1}D_{1}}}$ is $180^{\circ }$ because the facet $\bar{A_{1}B_{1}P_{1}}$ remains flat at the open and closed configurations. With the virtual crease, folding of the single-layer Yoshimura-ori structure can be treated as the rotation of rigid facets with respect to the elastic hinge-like creases. The obtained dihedral angles will be used to evaluate the mechanics of the Yoshimura-ori structure.

### Bending Kinematics

**FIGURE 4:**
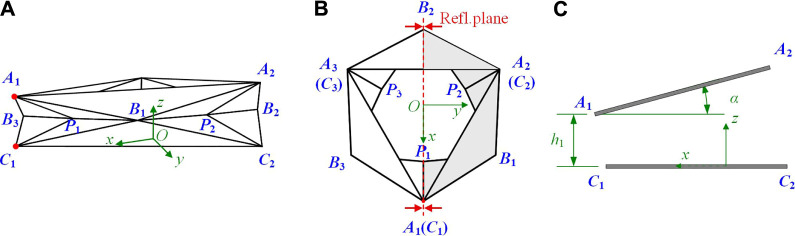
Geometry of a single layer Yoshimura-ori structure in bending motion. **(A)** A single-layer Yoshimura-ori structure when the distance between the vertices $A_{1}$ and $C_{1}$ is reduced. **(B)** Top-down view of the structure, where the shaded area represents half of the structure to be studied, and the reflection plane is denoted. **(C)** Schematic illustration of the variable α and $h_{1}$ during bending motion.

Bending motion is another important deformation pattern of the Yoshimura-ori structure. In this subsection, the bending motion of a single-layer Yoshimura-ori structure is examined, which is a prerequisite for evaluating the reachable workspace. Bending of a single-layer Yoshimura-ori structure can be achieved by reducing the distance between two vertices (e.g., $A_{1}$ and $C_{1}$). During the bending process, the fixed bottom plane $\bar{C_{1}C_{2}C_{3}}$ and the top plane $\bar{A_{1}A_{2}A_{3}}$ of the structure are assumed to be rigid. With the same *O*-*xyz* coordinate system (Figure 4A), the bending motion of a single-layer Yoshimura-ori structure can be described by the intersecting angle α between the planes $\bar{A_{1}A_{2}A_{3}}$ and $\bar{C_{1}C_{2}C_{3}}$ (Figure 4B) or the *z* coordinate of vertex $A_{1}$ (denoted by $h_{1}$). Through geometric analysis, the coordinates of $A_{1}$, $A_{2}$, and $A_{3}$ are given by:

$$A_{1}=\left[\sqrt{3}L/3+h_{1}\tan\left(\alpha /2\right),0,h_{1}\right],$$ (7a)

$$A_{2}=\left[\frac{\sqrt{3}L}{3}+h_{1}\tan\left(\frac{\alpha }{2}\right)-\frac{\sqrt{3}L}{2}\cos\alpha ,\frac{L}{2},h_{1}+\frac{\sqrt{3}L\cos\alpha }{2}\right],$$ (7b)

$$A_{3}=\left[\frac{\sqrt{3}L}{3}+h_{1}\tan\left(\frac{\alpha }{2}\right)-\frac{\sqrt{3}L}{2}\cos\alpha ,-\frac{L}{2},h_{1}+\frac{\sqrt{3}L\cos\alpha }{2}\right].$$ (7c)

For bending motion, the structure is only reflectional symmetric about the plane $\bar{A_{1}C_{1}B_{2}}$, while the $C_{3}$ rotational symmetry and the top-down reflectional symmetry no longer hold. Hence, for bending motion, half of the single-layer Yoshimura-ori structure (denoted by shade in Figure 4B) needs to be taken out for investigation.

During bending, constraint (3) still holds. To understand the bending behavior, the rigid-folding condition has to be broken by assuming that the lengths of the creases $\bar{P_{1}B_{1}}$, $\bar{P_{2}B_{1}}$, $\bar{P_{2}B_{2}}$, $\bar{P_{3}B_{2}}$, $\bar{P_{3}B_{3}}$, and $\bar{P_{1}B_{3}}$ are mathematically variable. Considering the reflection symmetry, the following constraints are applied

$$l_{\bar{P_{2}B_{1}}}=l_{\bar{P_{2}B_{2}}}=l_{\bar{P_{3}B_{2}}}=l_{\bar{P_{3}B_{3}}},\;l_{\bar{P_{1}B_{1}}}=l_{\bar{P_{1}B_{3}}}.$$ (8)

Solving Equations 3, 8 through numerical methods, the coordinates of vertices $B_{1}$, $B_{2}$, $B_{3}$, $P_{1}$, $P_{2}$, and $P_{3}$ at each step of folding can be expressed by αand $h_{1}$. Theoretically, the variables α and $h_{1}$ ($\alpha >0,\;h_{1}>0$) can be independent during bending motion. However, experimental observation indicates that they are partially interrelated. Hence, to reduce the numbers of variables, αand *h*_1_ are assumed to obey the following relation

$$\alpha \approx \alpha_{\max}-\frac{h_{1}}{h_{1\max}}\alpha_{\max},$$ (9)

Where $\alpha_{\max}$ is the maximum bending angle when $h_{1}$ reaches zero, and $h_{1\max}$ is a value of $h_{1}$ corresponding to zero bending angle. In this research, $\alpha_{\max}$ and $h_{1\max}$ can be obtained through geometric measurement of the prototype. Consequently, α is adopted as the independent variable to describe the bending motion, and the coordinates of all vertices can be expressed as functions of α. Particularly, if $\alpha_{\max}$ is assigned to be 0, the bending deformation model will degenerate into the axial deformation model discussed in Section 3.1.

**FIGURE 5:**
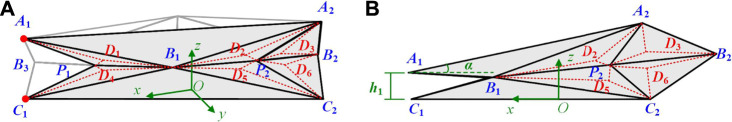
Virtual creases of a single-layer Yoshimura-ori structure under bending motion. **(A)** Panels A1B1P1, A2B1P2, A2B2P2, B1C1P1, B1C2P2, and B2C2P2 are respectively divided into two rigid triangular panels by a virtual crease. **(B)** Side view of the structure, where the variables α and h1 are denoted.

Similar to the axial kinematics, assuming variable lengths of the creases does not agree with the structure’s actual deformation. To evaluate the evolution of the dihedral angles during bending, virtual creases are added again. As shown in Figure 5, six virtual creases are introduced into the facets $\bar{A_{1}B_{1}P_{1}}$, $\bar{A_{2}B_{1}P_{2}}$, $\bar{A_{2}B_{2}P_{2}}$, $\bar{B_{1}C_{1}P_{1}}$, $\bar{B_{1}C_{2}P_{2}}$, and $\bar{B_{2}C_{2}P_{2}}$, respectively; each virtual crease divide the corresponding facet into two rigid panels. Referring to Equations 5, 8 and considering the symmetry, the positions of the virtual vertices $D_{i}$ (i=1,2...6) are constrained by:

$$l_{\bar{P_{1}D_{1}}}=l_{\bar{P_{2}D_{2}}}=l_{\bar{P_{2}D_{3}}}=l_{\bar{P_{1}D_{4}}}=l_{\bar{P_{2}D_{5}}}=l_{\bar{P_{2}D_{6}}}=\text{constant},$$ (10a)

$$l_{\bar{D_{1}B_{1}}}=l_{\bar{D_{2}B_{1}}}=l_{\bar{D_{3}B_{2}}}=l_{\bar{D_{4}B_{1}}}=l_{\bar{D_{5}B_{1}}}=l_{\bar{D_{6}B_{2}}}=\text{constant},$$ (10b)

$$l_{\bar{P_{1}D_{1}}}+l_{\bar{D_{1}B_{1}}}=l_{\bar{P_{1}D_{4}}}+l_{\bar{D_{4}B_{1}}}=l_{\bar{P_{2}D_{2}}}+l_{\bar{D_{2}B_{1}}}=l_{\bar{P_{2}D_{5}}}+l_{\bar{D_{5}B_{1}}}=l_{\bar{P_{2}D_{3}}}+l_{\bar{D_{3}B_{2}}}=l_{\bar{P_{2}D_{6}}}+l_{\bar{D_{6}B_{2}}}=\frac{L}{2},$$ (10c)

$$l_{\bar{A_{1}D_{1}}}=l_{\bar{A_{2}D_{2}}}=l_{\bar{A_{2}D_{3}}}=l_{\bar{C_{1}D_{4}}}=l_{\bar{C_{2}D_{5}}}=l_{\bar{C_{2}D_{6}}}=\text{constant}.$$ (10d)

Similarly, Equations 10a, 10b indicate that during folding, the distances from vertices $P_{1}$, $B_{1}$, $P_{2}$, $B_{2}$ to the newly introduced vertices $D_{i}\;\left(i=1,...,6\right)$ remain constant, Equation 10c implies that the corresponding sum equals to the initial distance between vertices $P_{1}$ and $B_{1}$ (or $P_{2}$ and $B_{2}$, equal to L/2), and Equation 10d suggests that the lengths of the virtual creases keep constant, which is determined from the open or closed configuration.

Combining Equations 7–10, the coordinates of all vertices can be determined at each step of bending deformation. Based on this, the dihedral angles between adjacent facets can also be calculated based on the law of cosines (exemplified in Equation 6). The obtained dihedral angles are preconditions for evaluating the mechanics of bending motion in Section 4.2.

### Reachable Workspace

**FIGURE 6:**
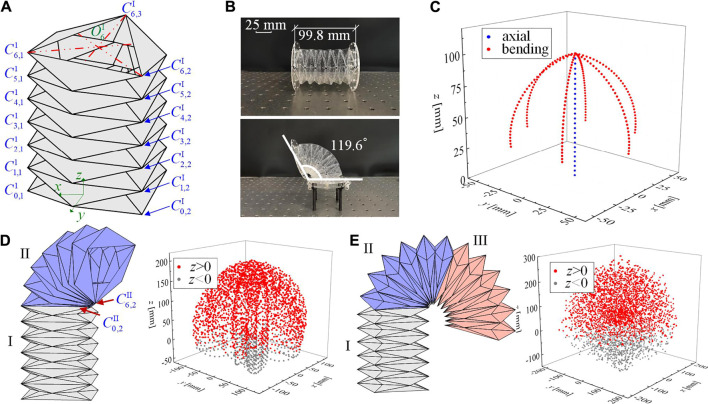
Geometry of a multi-layer Yoshimura-ori structure and its reachable workspace. **(A)** A six-layer Yoshimura-ori structure. **(B)** A six-layer Yoshimura-ori structure prototype. The top one displays a configuration without deformation, with its original length being 99.8 mm; the bottom one displays the configuration with the maximum reachable bending angle 119.6 °. **(C)** The reachable workspace of the six-layer Yoshimura-ori structure. **(D)** The geometry of a two-segment Yoshimura-ori structure and the corresponding reachable workspace. **(E)** The geometry of a three-segment Yoshimura-ori structure and the corresponding reachable workspace. The excellent 3D deformability is also demonstrated via experiment.

Based on the model of a single Yoshimura-ori layer, we then investigate the reachable workspace of the Yoshimura-ori structure without considering the effect of gravity. The reachable workspace is affected by the number of layers and the number of segments. Here, as an example, we first examine the reachable workspace of a six-layer Yoshimura-ori segment (Figure 5A). For describing purposes, the vertices of the structure are named as $C_{i,j}^{k}$ in this part (Figure 5A), where the subscript i indicates the layer number from the bottom up (j=0,...,6), the subscript j denotes the vertex number (i=1,2,3) in each layer, and the superscript k denotes the segment number (k=I,II,...). The established coordinate system remains the same as that in Figures 2–4, 6. Hence, for layer #1, a one-to-one correspondence between the coordinates of the vertices in Figure 4A and those in Figure 5A can be obtained

$${\left(\begin{array}{ccc} x_{C_{0,j}^{k}} & y_{C_{0,j}^{k}} & z_{C_{0,j}^{k}} \end{array}\right)|}_{j=1,2,3}={\left(\begin{array}{ccc} x_{C_{j}} & y_{C_{j}} & z_{C_{j}} \end{array}\right)|}_{j=1,2,3},$$ (11a)

$${\left(\begin{array}{ccc} x_{C_{1,j}^{k}} & y_{C_{1,j}^{k}} & z_{C_{1,j}^{k}} \end{array}\right)|}_{j=1,2,3}={\left(\begin{array}{ccc} x_{A_{j}} & y_{A_{j}} & z_{A_{j}} \end{array}\right)|}_{j=1,2,3}.$$ (11b)

In this research, the deformation of each Yoshimura-ori layer is characterized *via* the position of the endpoint, which locates at the centroid of the top equilateral triangle. During axial and bending motions, both the top and bottom equilateral triangles remain unchanged. Specifically, the coordinates of the centroid $O_{i}^{k}$ of the plate $\bar{C_{i,1}^{k}C_{i,2}^{k}C_{i,3}^{k}}$ yields

$$x_{O_{i}^{k}}=\frac{x_{C_{i,1}^{k}}+x_{C_{i,2}^{k}}+x_{C_{i,3}^{k}}}{3},y_{O_{i}^{k}}=\frac{y_{C_{i,1}^{k}}+y_{C_{i,2}^{k}}+y_{C_{i,3}^{k}}}{3},z_{O_{i}^{k}}=\frac{z_{C_{i,1}^{k}}+z_{C_{i,2}^{k}}+z_{C_{i,3}^{k}}}{3}.$$ (12)

Particularly, the coordinates of the endpoint $O_{6}^{\text{I}}$ is employed to characterize the reachable workspace of the six-layer Yoshimura-ori structure as a whole. Based on the assumption that the constituent layers’ bending angles are uniform, the coordinates of $O_{6}^{\text{I}}$ can be obtained *via* coordinate transformation

$$\left[x_{O_{i}^{k}},y_{O_{i}^{k}},z_{O_{i}^{k}}\right]=\left[x_{O_{i-1}^{k}},y_{O_{i-1}^{k}},z_{O_{i-1}^{k}}\right]T+\left[x_{O_{i-1}^{k}},y_{O_{i-1}^{k}},z_{O_{i-1}^{k}}\right],i=2,...6,$$ (13)

Where T is the coordinate transformation matrix

$$T=\left(\begin{array}{ccc} \cos\alpha & 0 & -\sin\alpha \\ 0 & 1 & 0\\ \sin\alpha & 0 & \cos\alpha \end{array}\right).$$ (14)

To make a quantitative evaluation, the value of the variable α and the height $h_{1\max}$ corresponding to zero bending angle are determined based on the observation of a six-layer Yoshimura-ori structure prototype (Figure 5B). Specifically, the angle α of a single layer is allowed to vary between $0^{\circ }$ and $20^{\circ }$($119.6^{\circ }/6\approx 20^{\circ }$), and $h_{1\max}$ is set to be 16.6 mm (99.8mm/6≈16.6mm). Figure 5C shows the theoretical workspace of a six-layer Yoshimura-ori structure. The blue dots denote the reachable positions achieved by axial deformation, which ranges from 0 to 100 mm. The red dots represent the reachable positions achieved by a point-to-point bending motion. Considering that the orthogonal projection of the Yoshimura-ori structure is a hexagon, point-to-point bending can be achieved along six directions, representing by the six red curved tracks in Figure 5C. The lowest point of each curved track is attained when the Yoshimura-ori structure is fully bent, with $\alpha =20^{\circ }$ for each layer.

If involving more segments into the structure, the reachable workspace can be expanded significantly. As an example, we analyze the reachable workspace of a Yoshimura-ori structure with two segments (each segment consists of six layers). The connection between segments is rigid so that the deformation of each segment remains independent. Figure 5D displays a configuration in which segment I is under axial deformation, and segment II is under bending deformation along vertices $C_{0,2}^{\text{II}}$ to $C_{6,2}^{\text{II}}$. With two segments, the furthest reachable distance of the endpoint $O_{6}^{\text{II}}$ will expand twice accordingly. Moreover, if the two segments deform differently, the combination of their deformations would let the endpoint $O_{6}^{\text{II}}$ reach new locations, thus, increasing the density of reachable positions in the workspace. Particularly, the endpoint could also reach the space with negative *z* values (gray dots). If the number of segments is increased to three, the furthest reachable distance of the endpoint $O_{6}^{\text{III}}$ will raise three times, and the reachable positions in the workspace would become denser.

## Folding Mechanics

To utilize the Yoshimura-ori structure as a robot segment, understanding its mechanical properties is also a necessity. In this section, theoretical derivations of the potential energy profile and the corresponding restoring force are carried out first. After that, quantitative evaluation of the restoring force is performed *via* experiments, rather than through theoretical efforts. This is because the stiffness of the creases and facets of the origami structure, as well as the stress-free configuration, is always unknown and is difficult to be accurately measured, and there is still a gap between the actual deformation mode and the assumed virtual-crease deformation mode. However, we remark here that the theoretical part is still meaningful. If the physical parameters can be obtained *via* inverse approaches (e.g., model-based identification (Liu et al., 2018) or data-driven identification (Liu et al., 2019)), the theoretical relations could serve as a useful tool for property prediction and optimization.

### Mechanics for Axial Deformations

With the virtual creases, folding of the Yoshimura-ori structure can be considered as rotations of rigid facets with respect to the elastic hinge-like creases. Hence, the potential energy of the structure with respect to folding can be evaluated. By assigning $k_{L}$ as the torsional stiffness per unit length of the creases, and $k_{B}$ as the torsional stiffness per unit length of the virtual creases (bending stiffness), the elastic potential energy of the structure can be determined, which is the sum of the potential energies originated from the creases and the virtual creases

$$\Pi_{\text{crease}}=\frac{k_{L}}{2}\left[\frac{4\sqrt{3}mnL}{3}{\left(\theta_{\bar{A_{1}B_{1}}}-\theta_{\bar{A_{1}B_{1}}}^{0}\right)}^{2}+4Ln\left(m-1\right){\left(\theta_{\bar{A_{1}A_{2}}}-\theta_{\bar{A_{1}A_{2}}}^{0}\right)}^{2}+\frac{\sqrt{3}nmL}{3}{\left(\theta_{{\bar{A_{1}P}}_{1}}-\theta_{\bar{A_{1}P_{1}}}^{0}\right)}^{2}\right],$$ (15a)

$$\Pi_{\text{virtual}}=2nmk_{B}l_{\bar{A_{1}D_{1}}}{\left(\theta_{\bar{A_{1}D_{1}}}-\theta_{\bar{A_{1}D_{1}}}^{0}\right)}^{2},$$ (15b)

$$\Pi_{\text{total}}=\Pi_{\text{crease}}+\Pi_{\text{virtual}}.$$ (15c)

In Equation 15, θ denotes the dihedral angles between adjacent facets, which have been derived in Section 3 and are functions of the height h; *n* and *m* represent the number of basal rectangles and the number of layers, respectively (Figure 1A). The stress-free configuration is defined as the open configuration (Figure 1B), and its corresponding dihedral angles are indicated by the superscript “0.” The potential energy profile can then be used to calculate the constitutive force-displacement relation by taking the derivative with respect to the displacement h, i.e.,

$$F=\frac{\text{d}\Pi_{\text{total}}}{\text{d}h}.$$ (16)

### Experiments on the Mechanics of Axial Deformation

**FIGURE 7:**
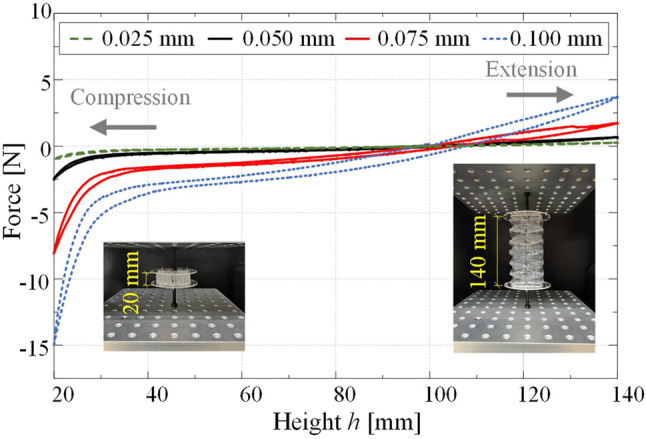
Experiments on the mechanical properties of a six-layer Yoshimura-ori structure prototype in the axial direction. The constitutive force-displacement curves are obtained *via* quasi-static axial compression and extension tests. The insets show two configurations of the prototype with h=20 mmand h=140 mm.

We then experimentally examine the mechanical behavior of the Yoshimura-ori structure during axial deformation. With the fabrication method introduced in Section 2, four 3×6 Yoshimura-ori structure prototypes are fabricated with PETE films of different thicknesses: 0.025, 0.05, 0.075, and 0.1 mm. For each prototype, three quasi-static compression and extension tests are performed (*see* the insets of Figure 7). The original length of the structure is 100 mm, we first compress it to 20 mm and then stretch it to 140 mm, with a loading speed of 0.5 mm/s. By averaging the three test results for each prototype, the corresponding force-displacement curve can be obtained (Figure 7). It shows that the constitutive force-displacement profile can be effectively tailored by adjusting the film thickness. With thicker film, the restoring force at the maximum-length configuration is much larger. For example, the restoring force grows more than 5.5 times from 0.67 to 3.72 N as the film thickness increases from 0.05 to 0.1 mm.

### Mechanics for Bending Motion

Similar to the scenario of axial deformation, the potential energy corresponding to the bending motion can also be derived *via* dihedral angles, lengths of creases, and torsional stiffness

$$\begin{array}{l} \Pi_{\text{crease}}=\frac{k_{L}}{2}\{\frac{2\sqrt{3}mL}{3}\left[{\left(\theta_{\bar{A_{1}B_{1}}}-\theta_{\bar{A_{1}B_{1}}}^{0}\right)}^{2}+{\left(\theta_{\bar{A_{2}B_{1}}}-\theta_{\bar{A_{2}B_{1}}}^{0}\right)}^{2}+{\left(\theta_{\bar{A_{2}B_{2}}}-\theta_{\bar{A_{2}B_{2}}}^{0}\right)}^{2}+...{\left(\theta_{\bar{C_{1}B_{1}}}-\theta_{\bar{C_{1}B_{1}}}^{0}\right)}^{2}+{\left(\theta_{\bar{C_{2}B_{1}}}-\theta_{\bar{C_{2}B_{1}}}^{0}\right)}^{2}+{\left(\theta_{\bar{C_{2}B_{2}}}-\theta_{\bar{C_{2}B_{2}}}^{0}\right)}^{2}\right]\\ +\frac{\sqrt{3}mL}{3}\left[{\left(\theta_{\bar{A_{1}P_{1}}}-\theta_{\bar{A_{1}P_{1}}}^{0}\right)}^{2}+...{\left(\theta_{\bar{C_{1}P_{1}}}-\theta_{\bar{C_{1}P_{1}}}^{0}\right)}^{2}+2{\left(\theta_{\bar{A_{2}P_{2}}}-\theta_{\bar{A_{2}P_{2}}}^{0}\right)}^{2}+2{\left(\theta_{\bar{C_{2}P_{2}}}-\theta_{\bar{C_{2}P_{2}}}^{0}\right)}^{2}\right]\\ +2L\left(m-1\right)\left[2{\left(\theta_{\bar{A_{1}A_{2}}}-\theta_{\bar{A_{1}A_{2}}}^{0}\right)}^{2}+...2{\left(\theta_{\bar{C_{1}C_{2}}}-\theta_{\bar{C_{1}C_{2}}}^{0}\right)}^{2}+{\left(\theta_{\bar{A_{2}A_{3}}}-\theta_{\bar{A_{2}A_{3}}}^{0}\right)}^{2}+{\left(\theta_{\bar{C_{2}C_{3}}}-\theta_{\bar{C_{2}C_{3}}}^{0}\right)}^{2}\right]\}, \end{array}$$ (17a)

$$\Pi_{\text{virtual}}=mk_{B}l_{\bar{A_{1}D_{1}}}\left[\begin{array}{l} {\left(\theta_{{\bar{A_{1}D}}_{1}}-\theta_{\bar{A_{1}D_{1}}}^{0}\right)}^{2}+{\left(\theta_{\bar{A_{2}D_{2}}}-\theta_{\bar{A_{2}D_{2}}}^{0}\right)}^{2}+{\left(\theta_{\bar{A_{2}D_{3}}}-\theta_{\bar{A_{2}D_{3}}}^{0}\right)}^{2}+...\\ {\left(\theta_{\bar{C_{1}D_{4}}}-\theta_{\bar{C_{1}D_{4}}}^{0}\right)}^{2}+{\left(\theta_{\bar{C_{2}D_{5}}}-\theta_{\bar{C_{2}D_{5}}}^{0}\right)}^{2}+{\left(\theta_{\bar{C_{2}D_{3}}}-\theta_{\bar{C_{2}D_{6}}}^{0}\right)}^{2} \end{array}\right],$$ (17b)

$$\Pi_{\text{total}}=\Pi_{\text{crease}}+\Pi_{\text{virtual}},$$ (17c)

Where the variables m, L, $k_{B}$, $k_{L}$ has been defined in Section 4.1. Note that during bending, the bending angle α is the independent variable. Therefore, all the dihedral angles, and hence, the total potential energy of the structure, can also be expressed as functions of α. However, it is worth pointing out that during quasi-static tests, bending of the 3×6 Yoshimura-ori structure is achieved by adjusting the distance d between vertices $C_{6,j}^{k}$ and $C_{0,j}^{k}$, which can also be expressed as a function of α (Figure 5A). Thus, the force for bending the structure can be obtained by:

$$T=\frac{\text{d}\Pi_{\text{total}}}{\text{d}\alpha }\cdot \frac{\text{d}\alpha }{\text{d}d}.$$ (18)

### Experiments on the Mechanics of Bending Deformation

**FIGURE 8:**
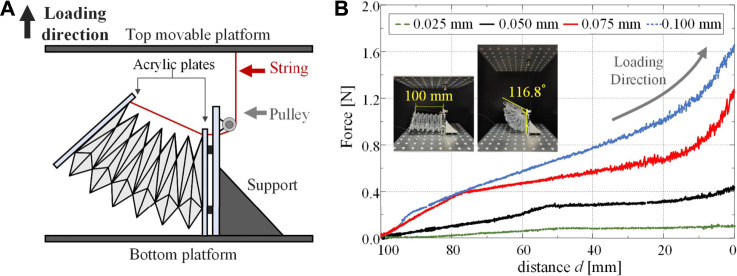
Experiments on the mechanical properties of a six-layer Yoshimura-ori structure prototype for bending deformation. **(A)** Schematic illustration of the setup for the equivalent bending test. **(B)** The obtained force-displacement curves for the four prototypes with different film thicknesses. Insets show the initial and final configurations of the prototype (i.e., d=100 mm and d=0 mm).

To experimentally examine the relation between the tension force and the bending motion of the Yoshimura-ori structure, a special setup is designed (Figure 8A). Specifically, the Yoshimura-ori structure is clamped between two acrylic plates; the right plate connected with the support is fixed with the bottom platform of the Instron universal testing machine. To measure the force required for bending deformation, an inextensible string is employed to bend the structure by reducing the distance between vertices $C_{0,j}^{1}$ and $C_{6,j}^{1}$. A pulley is fixedly installed on the support, and the string, which is threaded through the pulley, is connected with the left plate at the top vertex, and the other end of the string is connected with the top movable platform of the testing machine. The pulley could effectively eliminate the effect of friction. Hence, when the top platform is lifted, the distance between vertices $C_{6,j}^{\text{I}}$ and $C_{0,j}^{\text{I}}$ will be reduced, which equivalently bends the Yoshimura-ori structure (*see* insets in Figure 8A), and the required force for bending can be recorded accordingly. Bending tests are performed on four Yoshimura-ori structure prototypes with thicknesses 0.025, 0.05, 0.075, and 0.1 mm, and each prototype is tested three times. The original length of the prototype (i.e., the initial distance d between $C_{6,j}^{\text{I}}$vertices $C_{0,j}^{\text{I}}$ and) is 100 mm, and we will reduce it to 0 mm, with a loading speed of 0.5 mm/s. Averaging the three test results of each prototype, the corresponding force-displacement curve can be obtained. Figure 8B indicates that the maximum force is reached when the structure is fully bent (d=0). Moreover, the flexural rigidity (i.e., the slope of the curve) can be tailored by changing the film thickness. As the film thickness grows from 0.025 to 0.1 mm, the restoring force will increase more than 20 times from 0.08 to 1.64 N.

Study on the mechanics of Yoshimura-ori structure could provide useful guidelines for robot design and actuator selection. For robot development purposes, folding of the Yoshimura-ori structure has to be robust and consistent, and the restoring forces at the maximum-length and the maximum-angle configurations cannot exceed the actuation limit. These two requirements are sometimes contradictory. With thicker PETE film, the folding process will be more robust and consistent, but meanwhile, it needs a larger actuation force to deform axially or to bend, which will thus restrict the deformability. Hence, compromising is always necessary during robot design. In this research, the PETE film with thickness 0.05 mm is selected to fabricate the robot segment, which corresponds to reasonable magnitudes of the restoring force at the maximum-length and the maximum-angle configurations (0.69 and 0.45 N, respectively), and the obtained origami structure could exhibit robust and consistent folding deformations.

## Yoshimura-Ori-Based Earthworm Like Robot

Based on an in-depth understanding of the kinematic and mechanical properties of the Yoshimura-ori structure, we can then integrate the origami structure with axial and bending actuation mechanisms into an earthworm-like robot segment with excellent 3D deformability. Connecting multiple segments into a robot, an earthworm-like robot with 3D locomotion capability is expected.

### Design and Prototype of A Robot Segment

**FIGURE 9:**
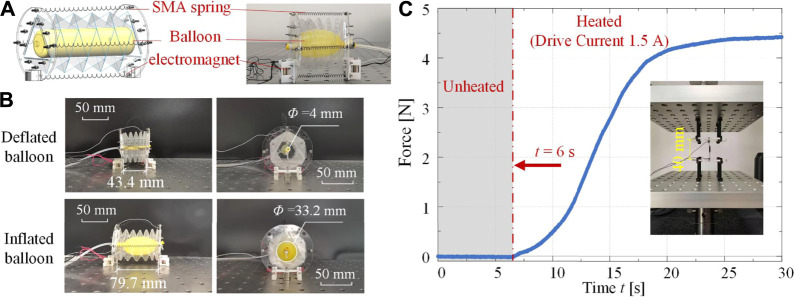
CAD design, prototype, and actuation mechanism tests of an earthworm-like robot segment. **(A)** The CAD design and the photo of a robot segment. **(B)** Axial actuation tests of a robot segment. By inflating the internal balloon, the segment switches from the axially-contracted state to the axially-extended state. Both the front view and the side view of the prototype are given. **(C)** The experimental actuation force profile of the SMA spring. The grey area represents the period when no current is applied, and the inset shows the test setup.

To acquire active 3D deformability, two types of actuators are incorporated into the robot segment: a pneumatic balloon for axial deformation and four SMA springs for bending deformation. Considering that the Yoshimura-ori structure cannot exhibit radial deformation during folding, additional bristle-like structures made of electromagnets are added to achieve anchoring effects. Figure 9A shows the CAD design of a single segment.

In detail, for each robot segment, a 3×6 Yoshimura-ori structure (Figure 1A) is clamped between two acrylic plates. The actuators, including the pneumatic balloon, the SMA springs, and the electromagnets, are also attached to the acrylic plates (Figure 9A). Details of these actuators are listed in Table A1 at the end of the paper. The balloon actuator locates at the axial axis inside the Yoshimura-ori structure and is controlled by pressure with high accuracy *via* the E/P pressure regulator. By increasing the pressure, the balloon will expand along the axial direction and extend the Yoshimura-ori structure, switching the robot segment into the axially-extended configuration; when the pressure is released, the balloon could provide sufficient force to pull the robot segment back to the axially-contracted configuration. Four SMA springs are set at the top, bottom, left, and right sides of the robot segment, and each SMA spring can be actuated independently. When applying electric current to an SMA spring, it will shorten quickly and thus bend the robot segment in the associated direction; when the current is removed, the SMA spring will be cooled and gradually return to its original length. By combining the deformations of the balloon and the SMA springs, the bending deformation can be achieved more effectively. Hence, the pneumatic balloon actuator is needed in both axial and bending deformations, while the SMA springs are only used in bending. Moreover, to facilitate robotic locomotion, electromagnets are installed at the bottom of the acrylic plates, which could anchor with magnetic media when it is energized.

Note that due to the introduction of actuation components, the kinematical properties of the robot segment will be different from the Yoshimura-ori structure segment, including the length of the prototype at the deformation-free state, the maximum bending angle, etc.

### Actuation Tests

Figure 9A also displays the photo of a robot segment prototype. It weighs only 29.8 g. The mechanical properties of the Yoshimura-ori structure have been comprehensively studied in Section 4. Here, the effectiveness of the actuators is demonstrated.

First, axial actuation of the robot segment through the pneumatic balloon is tested. The air pressure P of the balloon can be controlled precisely *via* the E/P Pressure Regulators (ITV ITV0030). In this research, the air pressure is set to be 0.162 MPa, which could effectively inflate the balloon. Figure 9B shows two configurations of the robot segment, one at the axially-contracted configuration with the internal balloon uninflated, and the other at the axially-extended configuration with the internal balloon inflated. Through inflation, the segment is extended from 43.4 to 79.7 mm, with a net deformation of 36.3 mm. The diameter of the internal balloon increases from 4 to 33.2 mm, which, however, does not affect the radial dimension of the robot segment. Note that the reachable maximum length is small than the original length of the Yoshimura-ori structure, this is due to the constraints from the SMA spring and to prevent the burst of the balloon.

Second, the mechanical properties of the SMA spring are evaluated. The SMA spring is fixed on the Instron universal testing machine and its initial length is set to be 40 mm. At t=6s, a current of 1.5 A is applied to heat the SMA spring and lasts for 24 s, during which, the contraction force is recorded. The test is performed three times, and the actuation force profile can be obtained by averaging the three test results (Figure 9C). It shows that the actuation force profile of the SMA spring is nonlinear in the overall trend. When there is no current (from 0 to 6 s), the actuation force remains zero; with the current input, the actuation force keeps increasing and reaches the maximum (4.39 N) at 30 s, which is larger than the maximum restoring force (0.46 N) for the bending motion of a single Yoshimura-ori segment. Hence, the SMA spring can provide sufficient force to help the Yoshimura-ori structure achieve bending.

### Demonstration of the 3D Deformability

**FIGURE 10:**
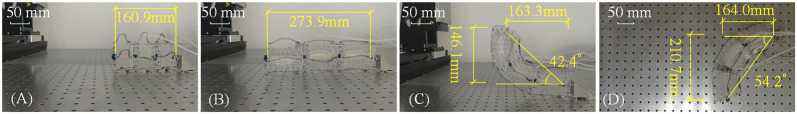
**(A–D)** Photos show the axial contraction, axial extension, up-contraction, and left-contraction of the three-segment Yoshimura-ori structure prototype.

To vividly demonstrate the 3D deformability, a prototype with three robot segments is tested. To test the deformability, one end of the prototype is fixed on the support; we use a camera to record the deformation process and measure the geometry. Figures 10A–D illustrates the axial contraction, axial extension, up-contraction, and left-contraction of the three-segment Yoshimura-ori structure prototype. At the axially-contracted and the axially-extended configurations, the axial length of the prototype is 160.9 and 273.9 mm, respectively. Hence, the net axial deformation of the three-segment prototype is 113.0 mm, which is 60.23% of its original length (160.9 mm). Such excellent axial deformability is desirable in earthworm-like robot development for achieving fast locomotion. Note that theoretically, the structure can be fully contracted to a very small height; while practically, its reachable axial deformation is restricted by the actuators. At the up-contracted and left-contracted configurations, the bending angle can be up to 42.4° and 54.2°, respectively, which is also favorable to robot development for achieving effective turning and rising motions. Note that the bending angle of the up-contracted configuration is smaller than that of the left-contracted configuration, which is induced by the effect of gravity.

### Robot Design and Prototype

**FIGURE 11:**
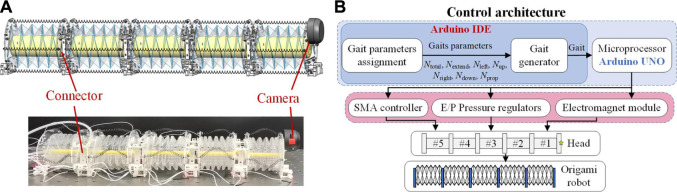
Prototype of a five-segment Yoshimura-ori-based earthworm-like robot and its control architecture. **(A)** CAD design and photo of the five-segment origami-based earthworm-like robot prototype. **(B)** Gait control architecture of the earthworm-like metameric robot.

Based on the designed robot segments a five-segment origami-based earthworm-like robot is prototyped by connecting five robot segments in series (Figure 11A). A camera is set at the head of the robot to capture the video of the working environment. The entire robot is 410 mm in length and 331.6 g in weight, and the weight-length ratio is only 0.81 g/mm. Comparing with other earthworm-like robots (e.g., 76.0 g/mm for the continuous meshworm robot (Boxerbaum et al., 2012), 1.39 g/mm for the earthworm-like robot with a spring-steel-belt body (Fang et al., 2014), and 1.27 g/mm for the underground earthworm-like explorer (Omori et al., 2009)), the robot has a very low weight-length ratio, which is beneficial for 3D locomotion.

Particularly, note that the weight of the robot forepart (consisting of a robot segment and the camera) is about 1.76 N, which is lower than the maimum actuation force of the SMA spring (4.39 N), indicating the feasibility for lifting the robot head. Moreover, the nominal attraction force of a single electromagnet is 10 N; with multiple embedded electromagnets and with a proper locomotion gait, the robot is possible to hold its weight (3.25 N), anchor with the magnetic media, and climb a wall, providing that the contact surface is not too smooth. However, further investigation on the locomotion performance by incorporating the robot weight, the electromagnet attraction force, and the friction coefficient is necessary, which is also an important part of our ongoing research.

### Locomotion Gait Design

Learning from the earthworm’s locomotion mechanism, i.e., the retrograde peristalsis wave, periodic gaits are proposed for the robotic locomotion. Fundamentally, a locomotion gait is defined as a sequence of the robot segment states with respect to the time, with the initial state being identical to the final state (Fang et al., 2014). Specifically, seven states of the robot segment are defined to construct the locomotion gait. Table 1 lists the seven states and the corresponding driving patterns.

**TABLE 1:** States and driving patterns of the robot segments.

States	Driving patterns
Axially-contracted	Deflate the balloon
Axially-extended	Inflate the balloon
Upward-contracted	Heat the upper SMA spring + Inflate the balloon
Right-contracted	Heat the right SMA spring + Inflate the balloon
Downward-contracted	Heat the lower SMA spring + Inflate the balloon
Left-contracted	Heat the left SMA spring + Inflate the balloon
Anchored	Energize the electromagnet

For the developed Yoshimura-ori based earthworm-like robot, seven parameters are needed to construct the locomotion gait, namely, the total number of the segments $N_{\text{total}}$, the number of the axially-extended segments $N_{\text{extend}}$, the number of the upward-contracted segments $N_{\text{up}}$, the number of the right-contracted segments $N_{\text{right}}$, the number of the downward-contracted segments $N_{\text{down}}$, the number of the left-contracted segments $N_{\text{left}}$, the number of actuated electromagnets $N_{\text{emagnet}}$ (anchored), and the number of propagating segments $N_{\text{prop}}$. These seven parameters are not independent, rather, they should satisfy the following constraints:

$$\begin{array}{l} N_{\text{extend}}\geq 0,N_{\text{up}}\geq 0,N_{\text{right}}\geq 0,N_{\text{down}}\geq 0,N_{\text{left}}\geq 0,\\ N_{\text{emagnet}}>0,N_{\text{prop}}>0,N_{\text{extend}}+N_{\text{up}}+N_{\text{right}}+N_{\text{down}}+N_{\text{left}}\geq 1,\\ N_{\text{total}}>2\left(N_{\text{extend}}+N_{\text{up}}+N_{\text{right}}+N_{\text{down}}+N_{\text{left}}\right)-N_{\text{prop}}, \end{array}$$ (19)

The segments with inflatable balloons ($N_{\text{extend}}+N_{\text{up}}+N_{\text{right}}+N_{\text{down}}+N_{\text{left}}$) are assumed to be gathered together, and they stay at the forepart of the robot at the initial state. To mimic the retrograde peristalsis wave of the earthworm, the actuation states (including the axially-extended, up/down/right/left contracted) are asked to propagate backward by $N_{\text{prop}}$ segments in each transition. In addition, there should always exist an anchoring point in the robot so that the other segments can lean against them and deform to achieve displacement. Hence, when the segments start to extend axially or to bend, at least one electromagnet in the rear of the actuated segments should be energized to achieve anchoring; when the actuated segments’ balloons start to deflate, the posterior electromagnets should be de-energized, while the anterior electromagnet of the front-most actuated segment should be energized so that the posterior segments can be pulled forward by the contraction of the balloons.

Figure 11B display the architecture of the robot gait controller, which consists of a computer (with Arduino Software IDE), Arduino UNO hardware, power supply, SMA controllers, and E/P Pressure Regulators. Inputting the seven gait parameters to the controller, admissible gaits of different locomotion can be generated. Gait signal will then be transmitted to voltage to the E/P Pressure Regulators, electromagnet, and SMA controllers, and finally actuate the SMA springs and balloon. However, the locomotion control of this robot is different from the traditional earthworm-like robot.

### Locomotion Tests

**FIGURE 12:**
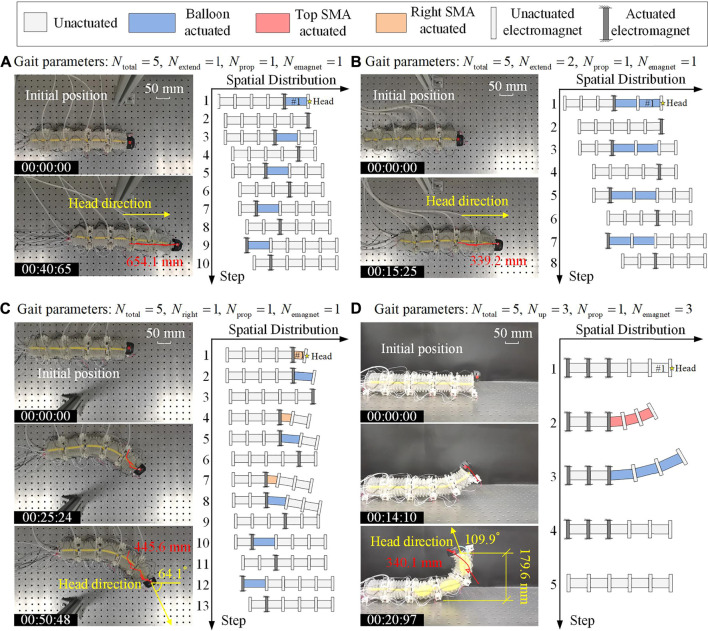
3D Locomotion tests, demonstrating *via* the snapshots and the extracted trajectory of the robot locomotion are demonstrated, as well as the corresponding locomotion gaits. **(A)** Rectilinear locomotion. **(B)** Rectilinear locomotion with another gait. **(C)** Right turning of the robot. **(D)** Rising up of the robot head. For these four locomotion tests, the adopted gait parameters are also listed.

Based on the robot prototype shown in Figure 11A and the gait controller provided in Figure 11B, locomotion tests are carried out on a horizontal iron plane. The robot’s locomotion is recorded by cameras, from which the trajectories of the robot head can be extracted and analyzed. To demonstrate the predicted 3D locomotion capability of the robot, Figure 12 shows the video snapshots of four locomotion tests, which include three types of locomotion, namely, rectilinear locomotion, turning locomotion, and rising locomotion.

Figure 12A (Supplementary Video S1) displays the snapshots for a rectilinear locomotion test and the corresponding gait (demonstrated as the state sequence of the robot segments). For the gait used in this test, each period of the robotic locomotion contains 10 steps, and only one segment is extended (i.e., the balloon is actuated) in every other step. Specifically, in the first step, the balloon in the head segment (segment #1) is pneumatized in 0.8 s, and the rear electromagnet of segment #1 is activated, which can push the robot head forward. In the second step, the front electromagnet of segment #1 is actuated, and the balloon of segment #1 is deflated in 1.1 s, which would pull the rest of the robot forward. In the following steps, actuation of the balloon and the electromagnet as a whole will propagate backward, generating a retrograde peristalsis wave, similar to the earthworm’s wave. With such a gait, the robot moves 327.1 mm in a period of 20.33 s, with an average speed of 16.09 mm/s. Figure 11A shows the snapshot at 40.65 s, where a 654.1 mm displacement is achieved by the robot. With five segments, the metameric robot could be equipped with more than one rectilinear locomotion gaits. Figure 12B (Supplementary Video S2) depicts another rectilinear locomotion with a different gait. The main difference is that two balloons are pneumatized in every other step. Hence, the robot needs only eight steps to finish a period, and the robot achieves faster locomotion with an average speed of 22.24 mm/s (i.e., move 339.2 mm in a period of 15.25 s).

In the third test, we demonstrate the planar locomotion, and the corresponding gait control is shown in Figure 12C (Supplementary Video S3). In this case, as an example, the three anterior segments are asked to perform right-contraction for turning purposes, and the two posterior segments are asked to perform axial extension for propelling purposes. In the first step, the right SMA spring of segment #1 is heated and contract in 4 s, and its rear electromagnet is energized so that the robot head turns to the right. In the second step, the rear electromagnet of segment #1 keeps energized, and the balloon of segment #1 is inflated to push the robot head forward right. In the third step, the anterior electromagnet of segment #1 is activated, and the balloon of segment #1 is deflated to pull the rest of the robot forward right. In the following steps, actuation of the balloon, the SMA spring, and the electromagnet as a whole will propagate backward. With such a gait, the robot can move 445.6 mm in two periods (50.48 s), with an average speed of 8.83 mm/s, and the robot head turns an angle of 64.1°.

The last test is the rising locomotion test, which is significantly distinct from the rectilinear and planar locomotion. For the gait used in this test, each period of the robotic locomotion contains five steps (Figure 12D (Supplementary Video S4)). In the first step, the last three electromagnets at the rear of the robot are energized to firmly anchor with the ground so that the robot can remain stable when the head is raised. In the second step, the top SMA springs of segments #1, #2, and #3 are heated and contract in 13 s, so that the robot head would be lifted. In the third step, the balloons in segments #1, #2, and #3 are inflated, which pushes the robot head upward. In the next step, the balloons are deflated and the robot head is lowered. Finally, all actuators are deactivated and the robot returns to its initial state. With such a gait, the robot can raise its head to 179.6 mm, and the rising angle could reach 109.9°.

## Discussion and Conclusion

Inspired by earthworms’ outstanding locomotion capability in various environment, earthworm-like locomotion robot has received extensive attention in many fields. While rich robot designs have been proposed for achieving rectilinear and planar locomotion, earthworm-like robots with excellent 3D locomotion capability are still immature. The inherent limitations in terms of the size, weight, and deformability of conventional earthworm-like robots greatly restrict the possibility of achieving 3D locomotion. As a result, aiming at expanding the locomotion capability from planar to spatial, an origami-based earthworm-like robot with unique 3D locomotion capability is designed and prototyped. The major innovation lies in the synthesis of the bio-inspired robot design principles, the extraordinary folding-induced origami kinematics, and the hybrid actuation mechanism. Among diverse origami patterns, the Yoshimura-ori structure stands out due to its simple crease pattern design, cylinder shape, and excellent axial and bending deformability, which is very similar to the bellow. However, the Yoshimura-ori structures possess some additional advantages that the bellows do not have. First, the Yohimura-ori structures have better designability due to the rich origami design space, and modification of the design can be achieved relatively easily by altering the crease patterns. Second, the Yoshimura-ori structure can be made of thin 2D materials (e.g. PETE film), which is very light in weight. On the other hand, the bellows used in robot are usually fabricated by 3D printing (Sheng et al., 2020) or silicone molding (Tang et al., 2020), which are more heavier in weight and diminish the 3D deformability of the robot.

After showing the geometry design and fabrication of the Yoshimura structure, we first establish an equivalent kinematic model of the single-layer Yoshimura-ori structure to understand its non-rigid folding motion. By assuming a uniform distribution of the bending and axial deformation in multiple Yoshimura-ori layers, we can then derive the reachable workspace of a six-layer Yoshimura-ori, which is made up of seven tracks (six curved tracks achieved by bending motion, and one straight track achieved by axial deformation). By increasing the number of segments, the furthest reachable distance of the endpoint can be significantly expanded, and the reachable positions in the workspace would become denser. Based upon the folding kinematics and by prescribing torsional stiffness to the creases, mechanical analyses are also performed to uncover the constitute force-displacement profiles during axial and bending deformations. Axial extension and bending tests indicate that the restoring forces for axial and bending deformations can be effectively tailored by changing the film thickness, which, therefore, provide necessary guidelines for robot design.

After understanding the kinematics and mechanics of the Yoshimura-ori structure, a five-segment earthworm-like locomotion robot is developed by synthesizing the origami structure and a hybrid actuation mechanism. Specifically, the Yoshimura-ori structure is made of 0.05 mm-thick PETE film, and the hybrid actuation mechanism consists of pneumatic balloons, SMA springs, and electromagnets. Before testing the five-segment robot, the effectiveness of a single robot segment in axial and bending deformations is first verified experimentally, and the extraordinary 3D deformability is demonstrated *via* a three-segment prototype. After that, the five-segment origami robot is evaluated. Comparing with conventional earthworm-like robots, the newly-developed robot prototype shows unique merits in terms of the weight-length ratio (which is only 0.81 g/mm) and the 3D deformation ability. To acquire 3D earthworm-like locomotion, gait control is proposed by learning from the earthworm’s retrograde peristalsis wave. Without loss of generality, various locomotion gaits can be generated by prescribing seven gait parameters. Experiments indicate that in addition to the rectilinear locomotion, the developed robot could perform effective left/right turning and rising locomotion, which, therefore, successfully enhancing the robot locomotion capability from 2D to 3D.

Overall, this research proposes a new type of earthworm-like locomotion robot based on the Yoshimura-ori structure. By integrating the excellent origami reconfigurability with the hybrid actuation mechanism, the obtained origami robot segment is featured with unique active 3D deformability, which, as a result, endows the earthworm-like robot with excellent 3D locomotion capability. The outcomes of this research could provide useful guidelines for the development of origami robots and worm-like locomotion robots. Note that a detailed mechanics model of the locomotion robot has not been established in this research, and the effects of gravity, payloads, and frictional resistance on the robot locomotion performance are not considered. These issues are of key importance in predicting and optimizing the robot performance, and they constitute a significant part of our ongoing work.

## Data Availability

The raw data supporting the conclusions of this article will be made available by the authors, without undue reservation.

## Appendix A. The Detailed Parameters of the Robot Segment’s Component

**TABLE A1:** The detailed parameters of the robot segment’s component.

Component	Size/model number
Balloon	Initial length: 70 mm
	Initial radius: 3.5 mm
	Initial thickness: 1.5 mm
SMA Spring (NiTi)	Initial length: 70 mm
	Outside diameter: 4.5 mm
	Wire diameter 0.5 mm
DC-powered electromagnet	Outside diameter: 12 mm
	Height: 12 mm
	Weight: 10 g
Acrylic plates	Thickness: 2 mm
E/P Pressure Regulators	ITV0030
Power supply	Auto Range DC Power Supply
	30 V/10 A
Arduino board	Arduino Mega 2560
